# 

**Published:** 1891-07

**Authors:** 


CONTENTS.

Vol. 38 No. 7.

| | PAGE. |
|---|---|
| Good Health | 145 |
| Coffee; its Use and Abuse | 147 |
| Telling the Truth for Fear of the "Cop" | 150 |
| The Mystery of a Lost Limb | 151 |
| Giants | 154 |
| Simple Diet the Safest | 155 |
| Fainting | 156 |
| Fidgety Parents | 156 |
| Convulsions in Children | 157 |
| Mental and Physical Work | 158 |
| Epidemic Influenza | 159 |
| Spring Tonics | 160 |
| Tendency to Physical Degeneration | 160 |
| Don't Shut up the Windows | 161 |
| Sunlight and Health | 161 |
| Bow-Legs | 162 |
| Sale of Tea in Russia | 162 |
| The Dandelion | 163 |
| Precious Stones; Early Notions regarding them | 163 |
| Miscellaneous | 164 |
| Elephantine Intelligence | 165 |
| Look for the "Russian Itch" | 166 |
| A Truth Worth Considering | 166 |
| Literary | 167 |
| Time | 168 |

INDEX TO ADVERTISEMENTS OF HALF A PAGE AND OVER.

| | PAGE. |
|---|---|
| Horsford's Acid Phosphate | I |
| Lydia E. Pinkham Med. Co. | II |
| Erie Medical Co. | IV |
| Ruckel & Hendel | V |
| Dr. J. C. Ayer & Co | V |
| Turf, Field & Farm Ass'n | VIII |
| J. H. Bunnell & Co | IX |
| Herba Vita | X |
| Royal Baking Powder | XI |
| I. S. Johnson & Co | XI |

